# Mycobacterial PstP impairs host RNA alternative splicing by dephosphorylation of spliceosome RBMX at S189

**DOI:** 10.1002/imo2.53

**Published:** 2025-01-09

**Authors:** Tianxian Liu, Jun‐Yu Xu, Lei Zhao, Yameng Fan, Shuyu Xie, Ke Ma, Ying Zhou, Minjia Tan, Bang‐Ce Ye

**Affiliations:** ^1^ Laboratory of Biosystems and Microanalysis, State Key Laboratory of Bioreactor Engineering East China University of Science and Technology Shanghai China; ^2^ State Key Laboratory of Drug Research, Shanghai Institute of Materia Medica Chinese Academy of Sciences Shanghai China; ^3^ Zhongshan Institute for Drug Discovery, Shanghai Institute of Materia Medica Chinese Academy of Sciences Zhongshan China

**Keywords:** inflammatory response, integrative multi‐omic analysis, mycobacteria, PLA2G7, RBMX S189, RNA alternative splicing, serine/threonine protein phosphatase PstP

## Abstract

*Mycobacterium tuberculosis* (Mtb) infection significantly alters host cellular signaling and protein functions, facilitating immune evasion and intracellular survival. However, the molecular mechanisms underlying these interactions remain incompletely characterized. Here, we employed a multi‐omics strategy, including proteomics, phosphoproteomics, transcriptomics and interactomics, to investigate the impact of Mtb infection on host cellular processes. Our study revealed that mycobacteria modulate RNA alternative splicing in host cells by reducing the phosphorylation levels within the spliceosome complex. We identified the serine/threonine protein phosphatase (PstP) as a key effector, dephosphorylating the spliceosome RNA‐binding motif protein (RBMX) at the serine 189 site (S189). This modification influences the alternative splicing of *PLA2G7*, which encodes platelet‐activating factor acetylhydrolase, resulting an increase in the mRNA levels of a transcript containing exon9 (*PLA2G7‐*exon9+). Importantly, PLA2G7 isoform encoded by *PLA2G7‐*exon9+, in contrast to the isoform lacking exon9, acquires the ability to potentiate inflammatory responses. Collectively, our findings not only provide a comprehensive view of Mtb‐induced host regulatory networks but also elucidate a role for PstP in controlling a critical mediator of alterative splicing during infection.

## Highlights

- Multi‐omics analyses were performed during Mtb infection of the host.
- Mtb infection affects host alternative splicing by dephosphorylating spliceosomes.
- Mtb PstP affects *PLA2G7* alternative splicing by dephosphorylating the S189 site of RBMX.

## Graphical abstract

During Mtb infection, the serine/threonine protein phosphatase PstP is secreted into host cells and directly dephosphorylate S189 on the spliceosomal RNA‐binding motif protein RBMX. Dephosphorylated RBMX promotes pathological alternative splicing of the immune gene platelet‐activating factor acetylhydrolase *PLA2G7*, leading to an increase in the expression of the exon9‐containing transcript (*PLA2G7*‐exon9+). While the host primarily produces the *PLA2G7* isoform lacking exon9 under normal/physiological conditions, Mycobacteria challenge disrupts RNA splicing machinery and raises the levels of *PLA2G7*‐exon9+, thus influencing immune processes.

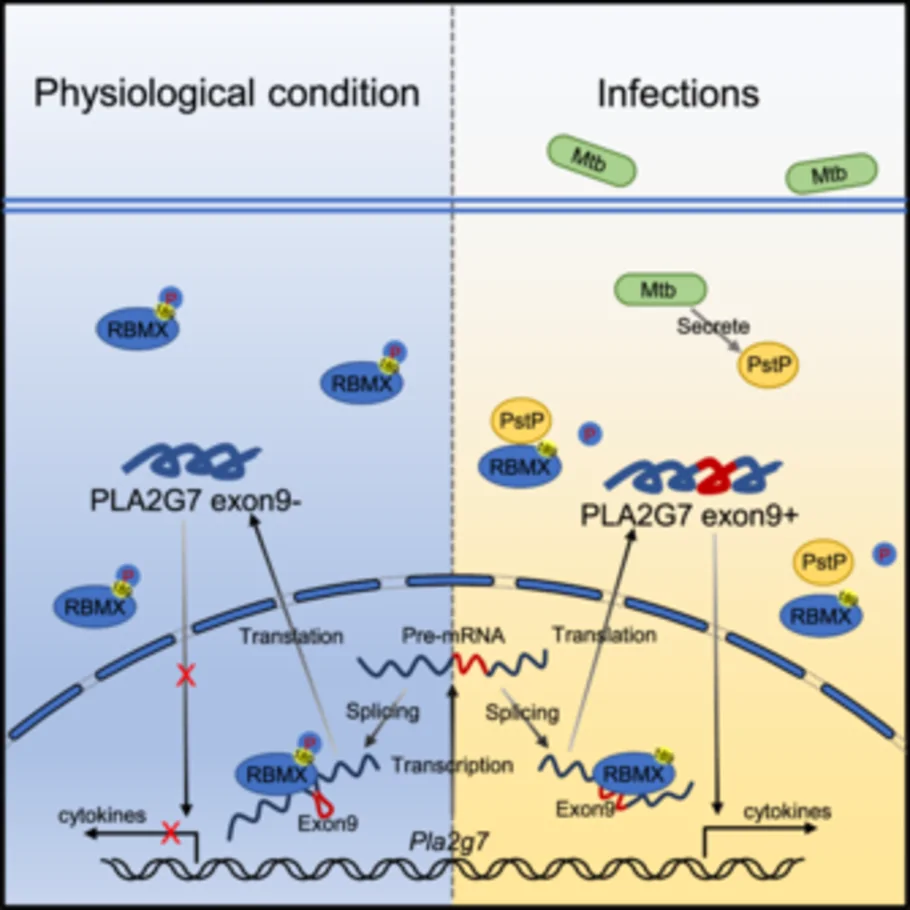

## 1 INTRODUCTION

Tuberculosis (TB), caused by *Mycobacterium tuberculosis* (Mtb), remains one of the leading causes of death worldwide. According to the World Health Organization (WHO), approximately 10.6 million people are infected with Mtb each year, resulting in 1.3 million deaths [1]. The emergence of drug‐resistant Mtb strains has significantly comprised the existing TB treatments, emphasizing the urgent need to uncover the mechanism of Mtb pathogenesis and host interactions. Understanding these mechanisms could facilitate the identification of novel therapeutic targets and the development of effective anti‐TB strategies [2].

Mtb is an intracellular pathogen that can survive and replicate within host macrophages for extended periods. To adapt to the nutrient‐limited and hostile environments of the macrophages, Mtb utilizes a variety of survival strategies, including hijacking the host cellular machinery, arresting phagosome maturation, and disturbing immune responses [3, 4]. Several secreted Mtb factors interact with host cytoplasmic or chromatin‐associated proteins, disrupting cellular signaling and gene expression, and ultimately contributing to pathogenesis [5]. Despite substantial progress, the molecular mechanisms underlying Mtb‐macrophage interactions remain poorly understood.

High‐throughput, unbiased omics approaches have been utilized to systematically characterize complex host‐pathogen interactions. Genome‐wide screens using RNA interference and CRISPR‐Cas9 have identified key host genes and pathways involved in infection and immune responses [6, 7, 8]. Furthermore, RNA sequencing (RNA‐seq) has revealed distinct gene expression profiles among the Mtb strains [9]. Proteomic methods, including immunoprecipitation coupled mass spectrometry (IP‐MS), have mapped protein–protein interactions (PPI) between Mtb‐secreted factors and host proteins, identifying virulence factors like the lipoprotein LpqN [10].

Posttranslational modifications (PTMs) play pivotal roles in the physiological alterations associated with mycobacterial infections. Protein phosphorylation, one of the most common PTMs, affects approximately 30% of the human proteome and is crucial for regulating signaling pathways. During Mtb infection, mycobacterial tyrosine phosphatases (PtpA and PtpB) and serine/threonine kinase (PknG) have been identified as secretory virulence factors that disrupt host signaling pathways [4]. Despite their relevance, only one quantitative phosphoproteomic study has investigated global phosphorylation dynamic in mycobacteria‐infected macrophages [11].

Previous studies have primarily focused on single‐omics approaches, which may overlook the integrative insights provided by multi‐omics approaches. To address this limitation, we employed a comprehensive approach to investigate the global proteome and phosphoproteome of THP‐1 macrophages exposed to Mtb and *M. smegmatis*. Our analyses revealed a significant enrichment of RNA splicing events among differentially phosphorylated proteins. Further RNA‐seq characterization identified dynamic alternative splicing events in immune‐related genes during infection. Through integrative protein interactomic and phosphoproteomic analyses, we identified that mycobacterial serine/threonine protein phosphatase PstP directly dephosphorylates serine 189 (S189) of the RNA‐binding motif protein (RBMX). This modification triggers pathological alternative splicing the platelet‐activating factor acetylhydrolase PLA2G7. These results provide new insights into the molecular mechanisms of TB pathogenesis and potential therapeutic targets.

## 2 RESULTS

### *Mycobacterium tuberculosis* H37Ra infection influences global protein phosphorylation and reduces spliceosome phosphorylation in the host

**FIGURE 1:**
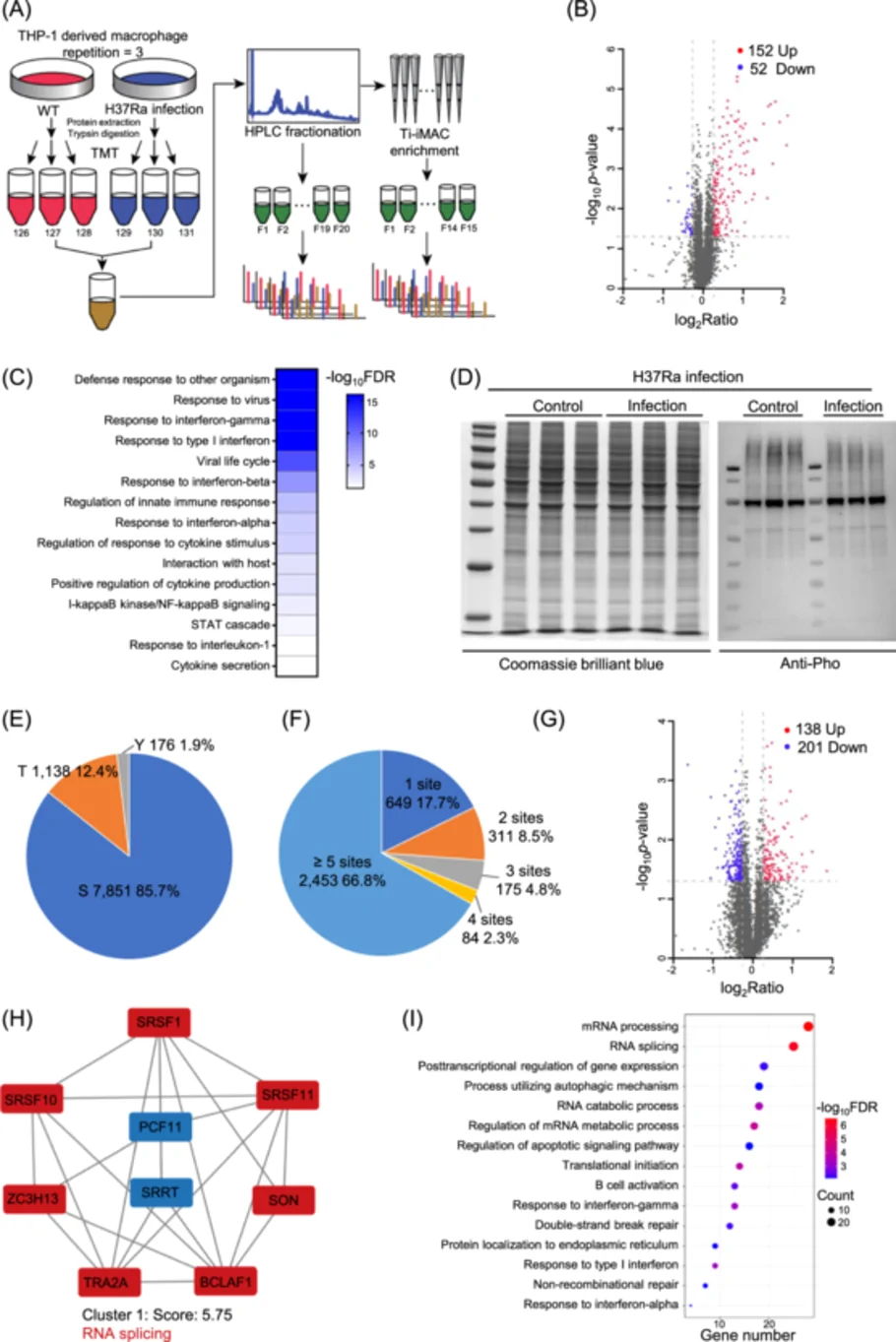
H37Ra infection affects host phosphorylation level. (A) THP‐1 cells were infected with Mtb H37Ra. After infection, cells were collected to label TMT‐6plex and then mixed to fractionate. Ti‐IMAC‐based phosphorylation enrichment was used for phosphoproteome. (B) Volcano plot of protein quantification after H37Ra infection (Infection/Control). (C) Gene Ontology biology process (GO‐BP) enrichment analysis of upregulated proteins after H37Ra infection. (D) Western blot analysis of global phosphorylation level by a pan phospho‐Ser/Thr antibody. (E) Number and ratio of identified phosphosites in S/T/Y. (F) Number and ratio of proteins containing 1/2/3/4/≥5 identified phosphosites. (G) Volcano plot of phosphoproteome quantification after H37Ra infection (Infection/Control). (H) Protein–protein interaction (PPI) analysis of phosphorylation down‐regulated proteins. Proteins in red are RNA splicing‐related proteins. (I) GO‐BP enrichment of phosphorylation down‐regulated proteins.

Pathogen infection can significantly alter host signal transduction and immune responses [12, 13, 14, 15, 16]. To investigate the effects of mycobacterial infection, THP‐1‐derived macrophages were infected with Mtb H37Ra at a multiplicity of infection (MOI) of 5. TMT‐based quantitative proteomic analysis was performed to explore the dynamic changes in the host proteome. (Figure 1A). A total of 6404 proteins were identified, among which 52 were significantly downregulated and 152 were upregulated (false discovery rate (FDR) < 0.05 and 1.2‐fold change; Figure 1B, Table S1). Enrichment analysis of the differentially expressed proteins showed that the upregulated proteins were mainly associated with host defense responses, host innate immune responses, cytokine responses, and cytokine‐related signaling pathways (Figure 1C and Table S2), consistent with previous studies [17]. Conversely, no significant pathway enrichment was observed among the downregulated proteins.

Pathogen proteins can affect host protein functions by modulating protein expression and posttranslational modification [18, 19, 20, 21, 22, 23, 24]. Host defense and immune responses rely on protein phosphorylation [19, 25]. We first performed western blot analysis to compare the global protein phosphorylation in THP‐1 cells with or without H37Ra infection. The result showed that the infected cells exhibited reduced phosphorylation levels of the proteins, particularly in the high‐molecular‐weight range (Figure 1D). To determine the differentially phosphorylated proteins in response to the infection, we performed a Ti‐IMAC‐based phosphopeptide enrichment assay (Figure 1A). This analysis identified 9165 phosphosites across 3672 phosphoproteins (Figure 1E and Table S3), with 85.7% (7851) occurring on serine, 12.4% (1138) on threonine, and 1.9% (176) on tyrosine residues (Figure 1F). Among these phosphosites, 138 were upregulated, and 201 were downregulated (FDR < 0.05 and 1.2‐fold change; Figure 1G and Table S3), indicating a global decrease in phosphorylation levels after H37Ra infection.

To elucidate the potential cellular implications of the decreased phosphorylation, we conducted protein–protein interaction (PPI) network analysis focusing on proteins with downregulated phosphosites (Figure 1H and Figure S1A). This analysis identified four distinct networks, with cluster 1 exhibiting the highest score (score 5.75, 9 proteins). Of these, seven proteins were associated with RNA splicing (Figure 1H). These results indicate that the observed reduction in protein phosphorylation may interfere with RNA splicing machinery in host cells, thereby impairing the host's capacity to regulate gene expression and respond to infection. To further validate this connection, we performed enrichment analysis of proteins with significantly altered phosphosites. Our results demonstrated that mycobacterial infection significantly affected mRNA processing, including RNA splicing (Figures 1I, S1B and Table S4). In addition, cell cycle‐related pathways were significantly enriched (Table S4), indicating that mycobacteria may interfere with cell cycle regulation.

Taken together, these results demonstrate that mycobacterial infection may disrupt host signaling pathways and cellular processes through selectively reducing protein phosphorylation. The downregulation of phosphorylation in proteins involved in RNA splicing suggests that mycobacteria may impact host defense by affecting this critical process. This is supported by previous reports indicating the importance of phosphorylation in spliceosome function [26, 27, 28, 29]. Additionally, the enrichment of cell cycle‐related pathways indicates that mycobacteria may broadly impact other essential host cellular processes.

### *Mycobacterium smegmatis* infection decreases phosphorylation of RNA splicing‐related proteins

To investigate the host response to mycobacterial infection, we used the faster‐growing bacteria *Mycobacterium smegmatis* MC2 155 (*M. smeg*) to infect the macrophages derived from THP‐1 cells with an MOI of 20, simulating a stronger infection. The infected cells were subjected to TMT‐labeled quantitative proteomic and phosphoproteomic analyses (Figure S2A).

The proteomic analysis revealed a pronounced response, identifying 7915 proteins, among which 1320 were upregulated and 1441 were downregulated (FDR <0.05 and 1.2‐fold change; Figure S2B, Table S5). Principal component analysis (PCA) demonstrated a clear separation between the control and infected groups (Figure S2C). Enrichment analysis of the upregulated proteins indicated activation of immune response and host defense pathways (Figure S2D). Conversely, the downregulated proteins were enriched in pathways related to cell cycle, organelle organization, and DNA replication, suggesting that severe infection disrupts essential cellular processes and cell division (Figure S2E, Table S6).

**FIGURE 2:**
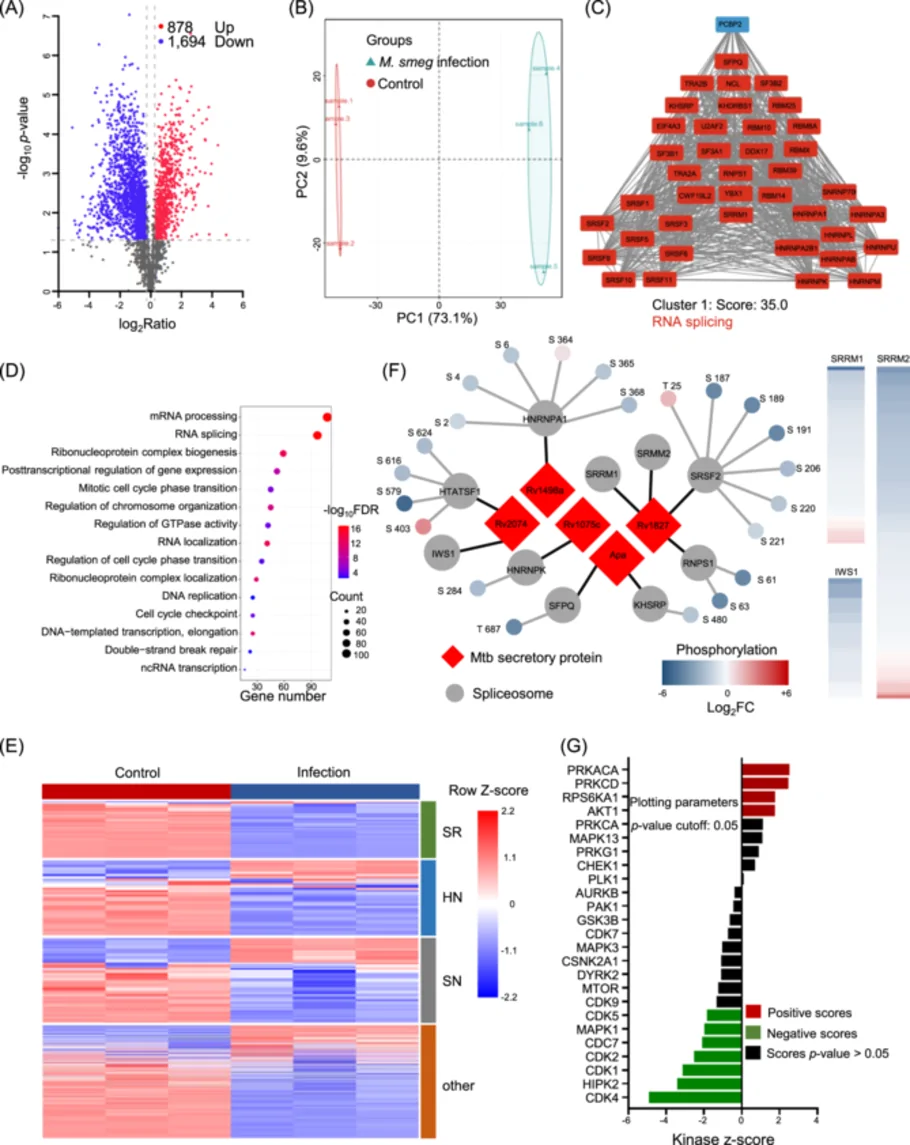
Mycobacterium smegmatis infection decreased phosphorylation and disturbed RNA splicing. (A) Volcano plot of phosphoproteome quantification after *M. smeg* infection (Infection/Control). (B) Principal component analysis (PCA) of replicates before and after infection. (C) PPI analysis of phosphorylation down‐regulated proteins. (D) GO‐BP enrichment of phosphorylation down‐regulated proteins. (E) Heatmap of phosphorylation level changes in RNA splicing‐related proteins after infection. SR: Serine/Arginine‐rich splicing factor; HN: Heterogeneous nuclear ribonucleoprotein; SN: Small nuclear ribonucleoprotein; other: Splicing‐related proteins but not including SR, HN, and SN. (F) Schematic representation of the interaction between host spliceosomes and Mtb secretory proteins from previous study. (G) Kinase substrate enrichment analysis (KSEA) of phosphorylated proteins.

Phosphoproteomics analysis demonstrated a significant change in phosphorylation, with 878 phosphosites upregulated and 1694 downregulated (Figure 2A, Table S7). These results are consistent with those observed in H37Ra infection, confirming that mycobacterial infection reduces host protein phosphorylation. Western blot analysis also revealed a global decrease in phosphorylation levels (Figure S2F). Furthermore, PCA confirmed clear distinctions between the control and infected samples (Figure 2B).

PPI network analysis identified 10 high‐confidence clusters (score ≥ 4), with cluster 1 showing the highest score (score 35.0, 40 proteins). Of these, 39 proteins were related to RNA splicing (Figure 2C, Figure S2G,H). Cluster 2 (score 18.4, 43 proteins) also included 11 RNA splicing‐related proteins (Figure S2G). Enrichment analysis of proteins with altered phosphosites ranked RNA splicing as the most affected pathway (Figure 2D, Table S8). Heatmap analysis demonstrated that RNA splicing‐related proteins exhibited significantly decreased phosphorylation, despite a slight increase in their total protein levels (Figure 2E, Figure S2I).

To further explore the connections between Mtb infection and the host RNA splicing machinery, we analyzed the interactions between host spliceosome‐related proteins and Mtb proteins using data from a previous study [10]. Five Mtb‐secreted proteins were found associated with host spliceosome components; most of the spliceosome showed a decrease in phosphorylation levels after infection (Figure 2F). However, no kinase or phosphatase activity has been reported for these Mtb proteins. Kinase substrate enrichment analysis (KSEA) demonstrated that activities of multiple kinases, including CDK1/2/4/5, HIPK2, CDC7, and MAPK1, were downregulated in the infection group (Figure 2G).

Overall, our study mimicked two phases of infection—mild and rapid severe—to characterize the host response. Our results demonstrate that mycobacterial infection decreases the phosphorylation of host RNA splicing‐related proteins, which may lead to impaired spliceosomal function and the subsequent RNA splicing process in the infected cells.

### Transcriptomic analysis reveals alterations in host RNA alternative splicing during mycobacterial infection

**FIGURE 3:**
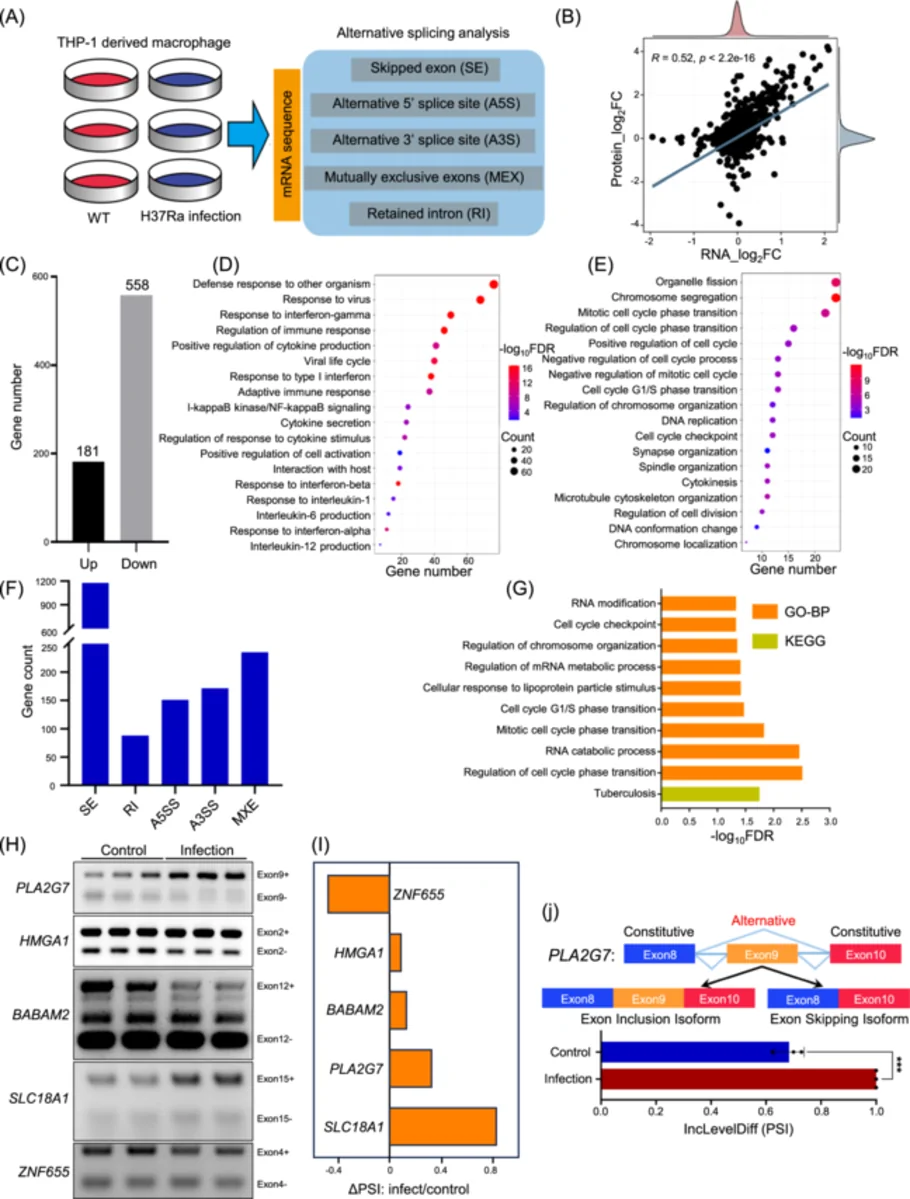
H37Ra infection transcriptomic analysis and RNA splicing verify. (A) Workflow of RNA‐seq and mRNA splicing analysis for H37Ra infection samples. (B) Transcriptomic and proteome correlation analysis of H37Ra infection samples. (C) Up and down genes number at the transcriptional level. GO‐BP enrichment analysis of (D) up‐regulated genes and (E) down‐regulated genes. (F) Abnormal splicing (AS) events in five splicing types of H37Ra infection transcriptomics. (G) GO‐BP and KEGG enrichment analysis of AS genes. AS events were picked up to (H) verify these AS ensure happened by reverse transcription‐polymerase chain reaction (RT‐PCR) and (I) ΔPSI of these genes in transcriptomics. (J) Schematic of alternative spliced PLA2G7 and the quantification results.

Proteomics and phosphoproteomics analyses have revealed that mycobacterial infections significantly affect host protein phosphorylation, particularly in proteins associated with RNA splicing. Rapidly severe infections may have a complex and comprehensive effect on splicing [30]. To determine whether these alterations impact RNA splicing events, we conducted RNA‐seq on Mtb H37Ra‐infected cells, enabling a comprehensive analysis of transcriptional changes associated with infection (Figure 3A).

First, we assessed the consistency between the transcriptomic and proteomic datasets. Pearson's correlation analysis showed a strong positive correlation average *r* = 0.52 with *p* < 2.2e−16 (Figure 3B). PCA demonstrated a clear separation between the transcriptomic profiles of control and infected groups, indicating significant transcriptional alterations caused by the infection (Figure S3A). A total of 11,924 genes were commonly expressed in both groups, while 409 and 481 genes were uniquely expressed in the control and infection groups, respectively (Figure S3B). Differential expression analysis identified 181 upregulated and 558 downregulated genes in response to infection (FDR < 0.05 and 1.5‐fold change; Figure 3C, Table S9). Furthermore, pathway enrichment analysis highlighted that the upregulated genes were primarily involved in host defense and immune responses (Figure 3D), whereas the downregulated genes were associated with DNA replication and cell cycle regulation (Figure 3E, Table S10). Notably, this pathway enrichment pattern closely mirrored the observations from the proteomic data (Figures 1C, S2D,E).

Next, we analyzed global splicing events using the rMATS method, a well‐described approach for detecting alternative splicing (AS) events [31]. Compared to the control, the infected samples exhibited 1820 abnormal AS events (rMATS, FDR < 0.05, and inclusion level differences ≥ 5%). Among these, skipped exons (SE, 1175) were the most frequently affected, followed by alternative 3′ splice sites (A3SS, 171), alternative 5′ splice sites (A5SS, 151), mutually exclusive exons (MXE, 235), retained introns (RI, 88) (Figures 3F, S3C and Table S11). Enrichment analysis of these abnormal AS events indicated a significant association with cell cycle‐related pathways (Figure 3G, Table S12), suggesting that Mtb infection disrupts RNA splicing in genes critical for cell cycle and cell division, thereby potentially influencing normal cell cycle progression. Moreover, KEGG pathway enrichment analysis identified tuberculosis as the only enriched pathway among abnormal AS events (FDR < 0.05, Figure 3G). These results indicate that RNA splicing plays a significant role in mycobacterial infection.

To validate splicing events altered during infection, we focused on genes with inclusion level differences ≥ 5% and FDR < 0.05 (Figure S3D), and used gene‐specific primers to distinguish canonical and alternatively spliced transcripts. Significant alteration in AS (ΔPSI > 5%) were observed in genes including *PLA2G7*, *HMGA1*, *BABAM2*, *SLC18A1*, and *ZNF655* (Figure 3H,I). These genes are related to the immune response, apoptosis, and cell cycle. Among these, *Pla2g7* was previously reported to regulate host metabolism and inflammatory responses [32, 33, 34]. Compared to the control, mycobacterial infection increased the level of exon9‐containing *PLA2G7* transcript by over 30% (Figure 3J).

Collectively, the transcriptomic analyses demonstrate that mycobacterial infection perturbs the alternative splicing of host genes related to immunity and cell cycle regulation. The observed splicing disruptions are likely driven by reduced phosphorylation of spliceosome proteins, as protein levels of spliceosome components remained unchanged.

### Mycobacterium protein phosphatase PstP regulates the alternative splicing of the host *PLA2G7* RNA

**FIGURE 4:**
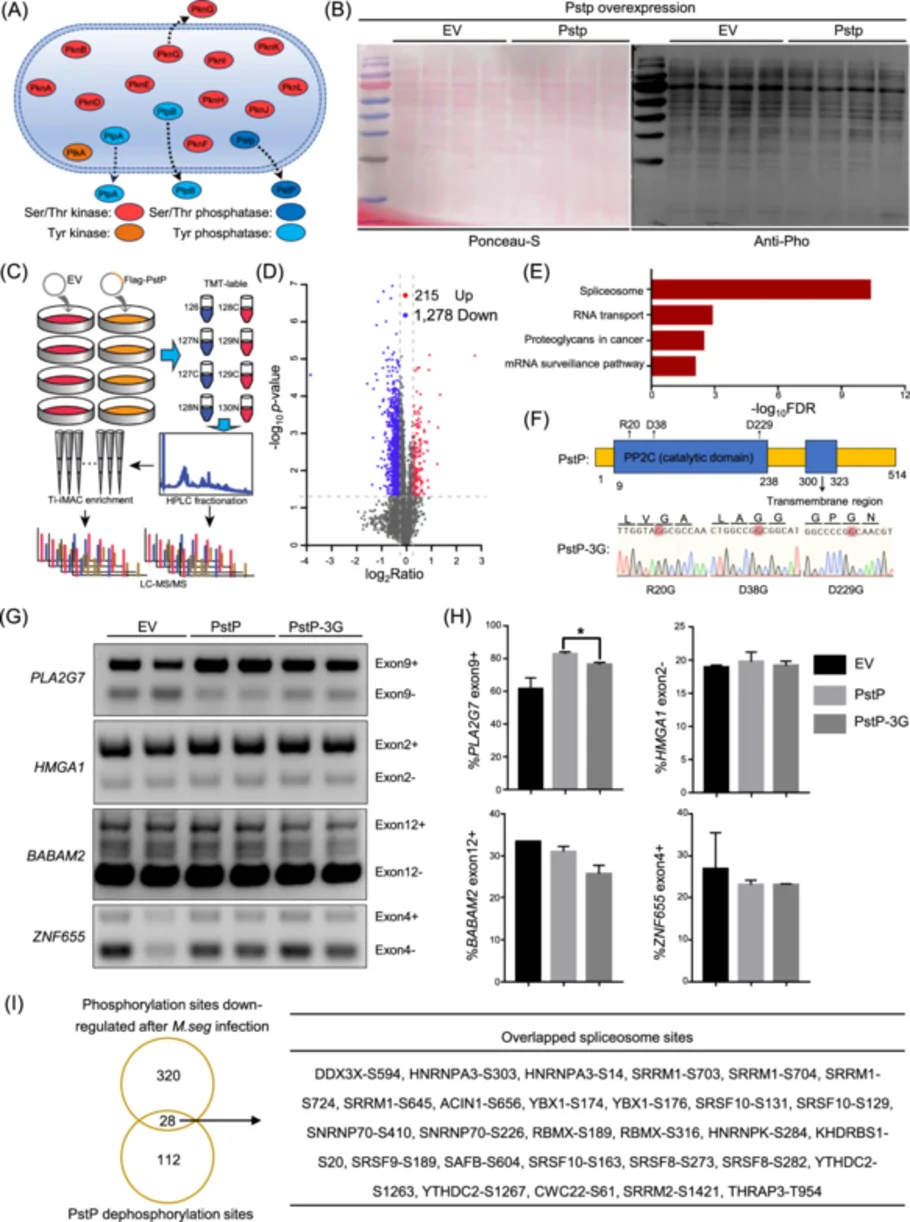
Mycobacterium Phosphatase PstP regulates host *PLA2G7* alternative splicing. (A) Schematic of Mycobacterium kinases and phosphatases. (B) Western blot analysis of global phosphorylation changes after overexpressed PstP in HEK‐293T cell line. (C) Workflow of PstP overexpressing quantification phosphoproteomics. PstP and EV were transfected to the HEK‐293T cell line, and these samples were labeled with TMT‐plex8. Following HPLC fractionation, a Ti‐IMAC‐based phosphorylation enrichment, and LC‐MS/MS were done to analyze the samples. Four replications per group. (D) Volcano plot of phosphoproteomics quantification after PstP overexpression (PstP overexpression/EV). (E) KEGG enrichment of phosphorylation down‐regulated proteins. (F) PstP functional domain and sites mutation from R20, D38, D229 to Gly to loss of phosphatase function. (G, H) RT‐PCR analysis of AS in HEK‐293T cell line transfected with EV, PstP or PstP3G. The results are presented as means ± SEM, *p < 0.05, **p < 0.01, ***p < 0.001. Two‐tailed unpaired Student's *t*‐test was used for statistical analysis. (I) Overlap of *M. smeg* infection down‐regulated and PstP overexpression down regulated splicing related proteins phosphosites.

The remodeling of host AS during infections has been widely reported [14, 26, 35, 36, 37, 38]; however, the underlying mechanisms remain unclear. Mycobacteria possess a complex phosphorylation system comprising 11 serine/threonine kinases, one tyrosine kinase, two tyrosine phosphatases, and a single serine/threonine phosphatase, PstP [39] (Figure 4A). PstP, a nonclassical secretory protein with eukaryotic‐like motifs, has been detected in Mtb culture medium supernatants [22, 40, 41]. Given that Mtb infection decreases host protein phosphorylation primarily on serine residues (Figure 1F), we hypothesized that PstP contributes to this effect.

To test this hypothesis, we overexpressed PstP in HEK‐293T cells and observed a significant reduction in global phosphorylation levels by western blot analysis (Figure 4B). We next examined whether PstP specifically targets spliceosomal protein phosphorylation. Using TMT‐labeled quantitative phosphoproteomic analysis (Figure 4C), we found nonsignificant changes in overall protein expression levels following PstP overexpression (Figure S4A, Table S13), suggesting that the observed effects were primarily driven by phosphorylation modulation rather than changes in protein abundance.

Phosphoproteomics identified 15,626 phosphosites, of which 215 were upregulated and 1278 were downregulated (FDR < 0.05 and 1.2‐fold change; Figure 4D, Table S14). Motif analysis of the dephosphorylated sites showed a conserved proline at the +1 position and neighboring serine or arginine residues, a motif characteristic of the SR domain in the spliceosome proteins (Figure S4B,C). Consistently, proteins with downregulated phosphosites were enriched in the spliceosome pathway, ranking at the top of pathway analysis (Figure 4E and Table S15). These results confirmed our hypothesis that PstP reduces phosphorylation of the spliceosome proteins, potentially affecting RNA splicing.

Previous studies have reported that mutations of Asp^38/229^ to Gly reduced the phosphatase activity of PstP by 90%, while a mutation of Arg^20^ to Gly reduced it by 60% [42]. To further evaluate the role of PstP, we generated a triple‐mutant PstP (PstP‐3G) combining these substitutions (Figure 4F). Functional analysis showed that ectopic expression of PstP in HEK‐293T cells significantly increased the production of the exon9‐containing transcript of *PLA2G7*. In contrast, the PstP‐3G mutant had little effect on this splicing event (Figure 4G,H). Notably, no changes in splicing were observed for *HMGA1*, *BABAM2*, or *ZNF655* under the same conditions.

To further understand the role of PstP in host protein phosphorylation remodeling, we compared the PstP‐downregulated phosphosites with those reduced during infections with H37Ra and *M. smegmatis*, respectively (Figure S4D, Figure 4I). Under the conditions of H37Ra infection and PstP overexpression, only two overlapping RNA splicing protein‐related phosphosites (SART1‐S448 and SRSF10‐S131) were identified (Figure S4D). The phosphorylation status of S131 in SRSF10 has been shown critical for *Bcl‐x* RNA splicing and subsequent modulation of cell apoptosis [27]. A greater overlap was found between the *M. smegmatis* infection and PstP overexpression, identifying 28 phosphosites across 17 splicing‐related proteins (Figure 4I).

Taken together, these results demonstrated that Mtb phosphatase PstP is a key regulator of host protein phosphorylation, significantly decreasing the phosphorylation levels, particularly in spliceosome‐associated proteins.

### PstP regulates *PLA2G7* alternative splicing by decreasing the RBMX S189 phosphorylation

We have shown that the Mtb phosphatase PstP affects *Pla2g7* AS by decreasing the phosphorylation of host spliceosomes. Through the infection and PstP overexpression phosphoproteomic analyses, we identified the spliceosome proteins regulated during infection and by PstP specifically (Tables S7, Figure 14). Among these, 28 spliceosome proteins were identified as candidates potentially regulated by PstP and influencing *PLA2G7* splicing (Figure 4I).

**FIGURE 5:**
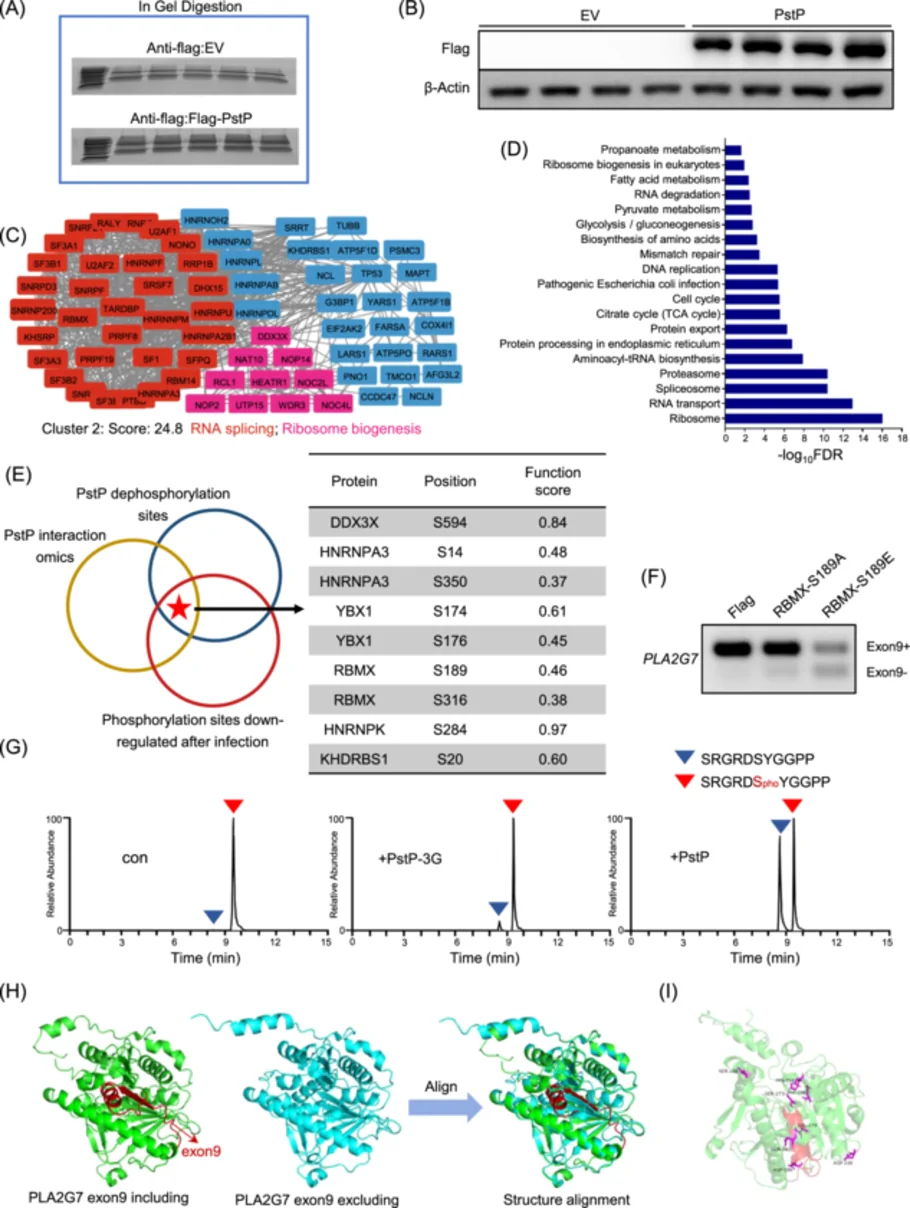
PstP regulates PLA2G7 AS by decreasing RBMX S189 phosphorylation level. (A) Schematic of PstP interaction proteins discovery. (B) Western blot to verify PstP overexpression. (C) PPI network of RNA splicing related cluster with score 24.8. (D) KEGG enrichment analysis of PstP interaction proteins. (E) The overlap of PstP interacting RNA splicing related proteins and phosphosites was also down‐regulated when overexpressed PstP or infected with *M. smeg*. (F) RT‐PCR to analyze PLA2G7 AS by overexpressing RBMX‐S189A and RBMX‐S189E. (G) Peptide dephosphorylation assay when added with PstP and PstP‐3G. (H) PLA2G7 exon9 including and excluding structures predicted by alphaFold2. (I) Functional amino acid residues affecting protein catalysis in PLA2G7.

To refine the list of targets, we conducted immunoprecipitation followed by mass spectrometry (IP‐MS) to define the PstP interactome. HEK‐293T cells were used to overexpress FLAG‐tagged PstP, and whole‐cell lysates were immunoprecipitated with anti‐FLAG beads and analyzed via in‐gel chromatography‐tandem mass spectrometry (LC‐MS/MS) (Figure 5A). Successful PstP expression in the cells was confirmed by western blot analysis (Figure 5B). Using label‐free intensity‐based absolute quantification (iBAQ) via MaxQuant, proteins identified exclusively in the PstP expressed samples within at least three biological replicates or with ≥twofold changes compared to the control (empty vector, EV) were considered as potential PstP interactors. A total of 1208 PstP‐interacting proteins were identified (present in ≥3 replicates, Figure S5A, Table S16) with good reproducibility. Protein enrichment and PPI analyses revealed significant enrichments in RNA splicing bioprocesses, ranked as the top two categories (Figures 5C,D, Figure S5B, and Table S17).

Next, to determine direct targets of PstP, we integrated data of the infection‐induced downregulated phosphosites, PstP‐overexpression downregulated phosphosites, and PstP interactors. This analysis identified nine candidate phosphosites across six splicing‐related proteins (Figure 5E), each scored for functional importance [43]. To validate whether phosphorylation at these sites regulates *Pla2g7* AS, we generated phosphor‐mimicking and phosphor‐dead mutants for the indicated proteins by replacing the Ser/Thr residues by Glu and Ala, respectively (Figure S5C–E). Among these, RBMX with a phosphor‐mimicking mutation at S189 (RBMX^S189E^) exhibited a remarkable inhibition on the exon9‐inclusion splicing of *PLA2G7* in HEK‐293T cells, while the corresponding phosphor‐dead mutant (RBMX^S189A^) showed no effect (Figure 5F). To confirm that PstP directly dephosphorylates RBMX^S189^, we synthesized the phosphorylated peptide corresponding to the residues 184–194 of RBMX. In vitro assays demonstrated that PstP effectively dephosphorylated this peptide, whereas PstP‐3G mutant almost lost this activity (Figure 5G). Overall, these observations demonstrate that Mtb phosphatase specifically targets RBMX S189.

Lastly, we performed structural predictions for PLA2G7 and its exon9‐excluded forms to assess potential changes in structure (Figure 5H). We observed no significant alterations in the overall protein structure, except in the exon9 excluding part, suggesting exon9 didn't affect the protein global conformation and may be located at the functional domain. To investigate the impact of exon9 deficiency, we labeled key activity residues on PLA2G7 as reported. As shown in Figure 5I, eight residues (Ser108, Ser273, Val279, Gln281, Asp286, Asp296, Asp338, and His351) [44, 45, 46] were reported affecting protein activity and four of them (Ser273, Val279, Gln281, and Asp286) located in exon9 which means exon9 deficiency could markedly affect the enzymatic activity of PLA2G7.

Taken together, these results suggest that RBMX‐S189 is a direct substrate of PstP and provides insight into how Mtb may influence host RNA splicing through dephosphorylation of spliceosome components.

### *PLA2G7* exon9+ but not exon9− variant enhances cellular inflammatory responses

**FIGURE 6:**
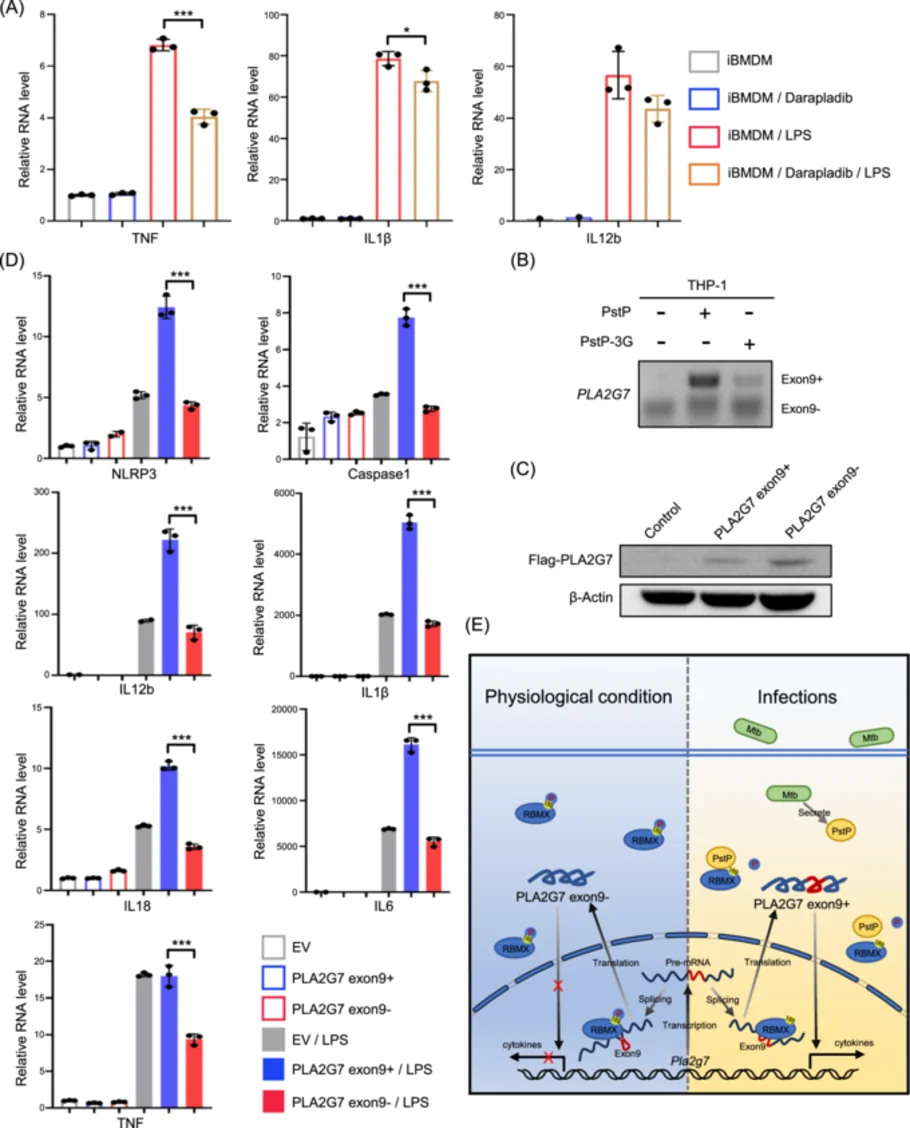
PLA2G7 exon9, including form, promotes inflammation response. (A) Inflammation factor transcriptional level after primed by LPS or added with PLA2G7 inhibitor Darapladib in iBMDM cell line. (B) RT‐PCR analysis of AS in THP‐1 cell line transfected with PstP and PstP‐3G. (C) PstP overexpression was verified in iBMDM cell line. (D) Inflammation factor transcriptional level in PLA2G7 exon9 including or excluding iBMDM cell line after primed by LPS or not. (E) Proposed model for Mtb infection affected host abnormal RNA splicing. Two‐tailed unpaired Student's *t*‐test (A, D) was used for statistical analysis. The results are presented as means ± SEM, *p < 0.05, **p < 0.01, ***p < 0.001. Two‐tailed unpaired Student's *t*‐test was used for statistical analysis.

PLA2G7 has recently been reported to regulate immunometabolism, with *Pla2g7* knockout shown to reduce NLRP3 pathway‐related inflammatory response in mouse [33]. Here, we demonstrated that treatment with Darapladib, the Lp‐PLA2 (encoded by *PLA2G7*) inhibitor, significantly decreased the TNF and IL1β transcriptional levels in LPS‐primed iBMDM cells (Figure 6A). Additionally, PstP overexpression THP‐1 cells potently influenced *PLA2G7* AS, increasing the levels of alternatively spliced exon9+ transcript (Figure 6B), confirming the role of PstP in modulating AS in immune cells.

The expression of both the exon9+ and exon9‐ splicing variants of *PLA2G7* was observed in HEK‐293T and Mtb‐infected THP‐1 cells (Figures 3H, 4G). However, it is unclear whether the proteins translated from these transcripts affect the inflammatory response. To address this, we overexpressed the *PLA2G7* exon9+ or exon9− variants in iBMDM cells, which were validated by western blot analysis (Figure 6C). We then assessed the expression of *Nlrp3*, *Caspase 1* and inflammatory cytokines including *Il12b*, *Il1ß*, *Il18*, *Il6*, and *Tnf*. The results indicated that only the *PLA2G7* exon9+ variant significantly enhanced the immune response (Figure 6D).

Based on our findings, we propose a model in which the expression of *PLA2G7* splicing variants is dynamically regulated under different cellular conditions. Under normal physiological conditions, immune cells predominantly express the exon9− variant, which does not appear to affect the host's inflammatory responses. However, during infection or other stress‐related states, the expression of the exon9+ variant is upregulated, actively modulating the host inflammatory responses (Figure 6E). These results suggest that the alternative splicing of Pla2g7 may serve as a key regulatory mechanism for fine‐tuning immune responses under varying conditions.

## 3 DISCUSSION

Mtb is an intracellular pathogen that manipulates host cellular processes through a repertoire of virulence factors, facilitating its survival and propagation [47, 48]. Our study integrates proteomics, phosphoproteomics, transcriptomics, and spliceomics to provide a comprehensive view of Mtb interactions with macrophages. Our data reveal a significant reduction in host protein phosphorylation levels following Mtb infection, with splicing‐related proteins excessively affected. These results demonstrate the ability of Mtb to disrupt normal host signal transduction and splicing regulation, likely contributing to its pathogenic success [38].

Alternative splicing plays a crucial role in regulating gene expression and generating protein diversity in eukaryotic cells. To further understand Mtb‐induced splicing alterations in the host, we conducted an in‐depth transcriptome analysis, identifying immunometabolism regulatory protein as a critical target. We demonstrate that Mtb infection induces AS of *PLA2G7*, resulting in increased levels of an exon9+ transcript, regulated by the phosphorylation state of RBMX at S189, a site targeted by the Mtb phosphatase PstP. While PLA2G7 has been widely studied as an immunometabolic regulator [32, 33, 34, 49, 50], this work is the first to examine its AS during infection. Interestingly, we discovered that only PLA2G7 exon9+ variant drives inflammatory responses, while exon9− variant excluding does not.

The Mtb kinase and phosphatase system contains 11 Ser/Thr protein kinases (STPKs), two Tyr phosphatases (PtpA and PtpB), and one Ser/Thr phosphatase (PstP), a remarkable extensive array for bacterial species [39]. These enzymes are secreted through classical or nonclassical pathways [22, 41], where they interfere with host cellular signaling. For example, PknG inhibits autophagosome maturation to sustain intracellular survival of Mtb [51], while PtpA dephosphorylates JUN and P38 to suppress TNF‐α transcription and regulates H3R2 methylation to modulate host gene expression [20, 25, 52]. Similarly, PtpB dephosphorylates PI3P to inhibit cell pyroptosis, promoting pathogen survival [22]. Despite its critical role, PstP remains less explored [41, 53]. Here, we demonstrate for the first time that PstP dephosphorylates RBMX at S189, modulating *PLA2G7* AS and linking its phosphatase activity to host immune responses.

Beyond phosphorylation, PTMs such as acetylation, propionylation, succinylation, lactylation, methylation, ubiquitylation, and phosphorylation also mediate Mtb‐host interactions [54, 55, 56, 57]. Ubiquitination has been widely reported during Mtb infection [19, 58, 59], with host‐conjugated ubiquitin activating virulence proteins, such as the dephosphorylation activity of PtpB [22]. In contrast, acetylation and phosphorylation mostly affect host proteins and are perturbed by Mtb‐secreted enzymes. For instance, the N‐acetyltransferase Eis inhibits JNK‐dependent autophagy and stimulates TNF‐α and IL‐6 production [60]. Our study corroborates earlier findings Mtb infection diminishes host protein phosphorylation [11] and extends these observations by identifying downregulated RNA splicing‐related phosphorylated proteins. This disruption of spliceosome function suggests a targeted mechanism through which Mtb modulates RNA processing to adapt to host environments.

Alternative splicing is frequently implicated in numerous diseases including cancer, metabolic disorders, and infections, and is a tightly regulated process influenced by various factors [14, 26, 27, 36, 38, 61]. In this study, we revealed that Mtb phosphatase PstP regulates *PLA2G7* splicing by dephosphorylating RBMX at S189. Intriguingly, the exon9‐ variant of *PLA2G7* is predominantly expressed under physiological conditions and has no effect on immune responses to infections. During infection, the pathogen‐induced shift toward the generation of exon9+ variant, enabling the host to produce a more robust inflammatory response.

## 4 CONCLUSION

This study provides a comprehensive overview of the global proteome and phosphoproteome changes in macrophages during infection with Mtb and *M. smegmatis*. We observed that RNA splicing events are significantly enriched among altered phosphoproteins, indicating the role of splicing regulation in the host response to infection. Through RNA‐seq, we identified dynamic AS events in immune‐related genes. Combining protein interaction and phosphoproteomic data, we determined that the mycobacterial serine/threonine protein phosphatase PstP directly dephosphorylates RBMX at S189, leading to a splicing alteration in *PLA2G7*. This change increases the production of an exon9‐including form of *PLA2G7*, which promotes the production of inflammatory factors.

## 5 METHODS

### Cell lines and bacterial strains

HEK‐293T cells were cultured in DMEM supplemented with 10% fetal bovine serum (FBS). THP‐1 and iBMDM cells were cultured in RPMI‐1640 medium supplemented with 10% FBS and 10 mM HEPES. All cell cultures were maintained in the presence of 100 mg/mL streptomycin and 100 U/mL penicillin at 37°C in a 5% CO_2_ incubator. iBMDM or iBMDM‐PLA2G7 stable expression cells were cultured in six‐well plate for 24 h and then treated with 100 ng/mL Darapladib for 6 h, followed by priming with 1 µg/mL LPS for an additional 4 h.

Mtb strain H37Ra was purchased from Gene Optimal and *M. smegmatis* MC2 155 strain was preserved in our laboratory. Both were grown in Mycobacterium complete medium (Gene Optimal) at 37°C.

### Reagents

The reagents used in this study included pan Phospho‐Serine/Threonine rabbit pAb (AP0893, ABclonal), anti‐FLAG (A8592, Sigma Aldrich), anti‐β‐actin (5125s, Cell Signaling Technology), liposomal transfection reagent (40802ES03, Yeasen), polybrene (40804ES76, Yeasen), trypsin (HLSTRY001C, Hualishi Scientific), mycobacterium complete medium (GOMY0026, Gene Optimal), darapladib (HY‐10521, MCE), anti‐FLAG M2 (A2220, Sigma‐Aldrich), reverse transcriptase (RK20429, ABclonal), phorbol‐12‐myristate‐13‐acetate (PMA) (P6741, Solarbio), and qPCR mix (RK21203, ABclonal).

### Mycobacterial infection

THP‐1 cells were seeded in 15 cm culture dishes, cultured for 2 days, and primed with 100 ng/mL PMA for 24 h. Cells were then cultured in fresh RPMI‐1640 medium for 12 h, infected with H37Ra (MOI = 5) or *M. smeg* (MOI = 20) for 2 h, washed with PBS, and incubated in fresh RPMI‐1640 medium containing 25 µg/mL gentamycin for 24 h before further analysis. Experiments were performed in triplicate.

### Protein extraction and MS sample preparation

THP‐1 derived macrophages were washed with ice‐cold PBS for three times and lysed with buffer (8 M urea in 100 mM NH_4_HCO_3_, 4× phosphatase inhibitor, 2× protease inhibitor cocktail). Lysates were sonicated (2 s on, 3 s off) for 3 min and centrifuged at 21,000 *g* for 10 min. The supernatants were quantified using a bicinchoninic acid (BCA) protein quantification kit (P0011, Beyotime).

Proteins were reduced with 5 mM dithiothreitol (DTT) at 56°C for 30 min and subsequently alkylated with 15 mM iodoacetamide (IAA) in darkness for 30 min. The reaction was terminated with 15 mM DTT for 30 min. Then the samples were diluted to 2 M urea concentration using 100 mM NH_4_HCO_3_ and digested with trypsin (1:50 [w/w]) for 16 h at 37°C, followed by a second trypsin digestion (1:100 [w/w]) for 4 h. Peptides were desalted with Sep‐Pak C18 columns and dried using SpeedVac.

### Tandem mass tagging (TMT) labeling

The desalted peptides were labeled with TMT6plex or TMT10plex. For H37Ra control/infection and *M. smeg* control/infection samples, peptides were labeled with 126‐131 channels using TMT6. For PstP overexpression samples, peptides were labeled with 126‐130C using TMT10. TMT reagent was dissolved in 15 μL ACN and then added to 80 μL TEAB buffer (50 mM, pH 8.5–9) containing 400 µg peptides. The reaction was performed at 25°C for 1 h, and then quenched with 0.4% hydroxylamine at room temperature for 15 min. The samples from each channel were mixed 1 μg to determine the labeling efficiency. After the labeling efficiency was more than 98%, the labeled samples were mixed and then desalted using Sep‐Pak C18 columns and dried by SpeedVac.

### High‐performance liquid chromatography (HPLC) fractionation

Labeled peptides were fractionated using HPLC with a Waters XBridge Prep C18 column (Waters Corp.) (19 by 150 cm). The flow rate was 1 mL/min. Samples were combined to 20 fractions for protein quantification and H37Ra infection samples/*M. smeg* infection samples/PstP overexpression samples were combined to 15/12/10 fractions for phosphoproteome quantification. The combined fractions were dried by a SpeedVac and used for further phosphorylated peptides enrichment.

### Phosphorylated peptides enrichment

Peptides were dissolved in 200 μL loading buffer (80% ACN, 6% TFA) and then loaded into a manually packed Ti‐IMAC microspheres tip chelated by Ti^4+^ for two times. The microspheres were then washed with Washing Buffer 1 (50% ACN, 6% TFA and 200 mM NaCl) two times and Washing Buffer 2 (30% ACN, 0.1% TFA). Phosphorylated peptides were eluted with Elution Buffer 1 (10% ammonium hydroxide) followed by Elution Buffer 2 (40% ACN, 10% ammonium hydroxide). The enriched peptides were then desalted, dried, and used for MS analysis.

### LC‐MS/MS analysis of TMT‐labeled samples

The LC‐MS/MS analysis was performed as described previously with minor modifications [62]. Briefly, samples were dissolved in solvent A (0.1% formic acid and 2% acetonitrile in water) and injected into a manually packed reverse‐phase C18 column (10 cm length × 75 μm inner diameter; C18 resin with 3 μm particle size; 90 Å pore diameter; Dikma Technologies Inc.) connected to a nano‐HPLC system (Thermo Fisher Scientific). The samples were analyzed at a flow rate of 300 nL/min.

For TMT labeling proteome, a 70‐min gradient was applied as follows: 2%–8% solvent B for 1 min, 8%–15% solvent B for 20 min, 15%–38% solvent B for 38 min, 38–47% solvent B for 5 min, 47%–80% solvent B for 1 min, 80% solvent B for 5 min. For H37Ra infection samples, Orbitrap Fusion mass spectrometer (Thermo Fisher Scientific) was used with a nanospray ion source in the positive mode. The mass resolution was 60 K at m/z of 200 for both MS1 and 15 K for MS2. For MS1, a m/z range of 450‐1, 650 was scanned with either a single charge or more than six charge discarded. The AGC targets were set as 5E5. For MS2, ions with an intensity larger than 50,000 were isolated and sequentially fragmentized by higher collision dissociation (HCD) with normalized collision energy of 40%. The dynamic exclusion duration was set to 50 s. For *M. smeg* infection samples and PstP overexpression samples, analyses were conducted using a Q Exactive HF‐X mass spectrometer (Thermo Fisher Scientific).

For TMT labeling phosphoproteome sample, the gradient was 110 min with 2%–30% solvent B for 90 min. Orbitrap Fusion mass spectrometer (Thermo Fisher Scientific) was used with a nanospray ion source in the positive mode. The mass resolution was 60 K at m/z of 200 for MS1 and 15 K for MS2. For MS1, a m/z range of 350–1800 was scanned with either a single charge or more than six charge discarded. The AGC targets were set as 5E5. For MS2, the ions with intensity larger than 50,000 were isolated and sequentially fragmentized by HCD with normalized collision energy of 40%. The dynamic exclusion duration was set to 25 s. For *M. smeg* infection samples and PstP overexpression samples, Q Exactive HF‐X mass spectrometer (Thermo Fisher Scientific) was used.

### Mass spectrometry data analysis

The raw MS data were analyzed using MaxQuant (version 2.0.1.0) with the following parameters: the database was downloaded from Uniprot (downloaded on 23/11/2021); the enzyme was set as trypsin/P with a maximum of two missed cleavages allowed. Variable modifications included oxidization(M), acetylation at the protein N‐terminus, the carbamidomethylation (C) was set as a fixed modification. For phosphoproteome analysis, phosphorylation (STY) was set as a variable modification. TMT6‐based MS2 reporter ion quantification was selected. The false discovery rate (FDR) was set to 1% at the protein, peptide, and modification levels. Phosphorylated sites with localization score below 0.75 were excluded from the phosphoproteome data. Phosphorylation data were normalized to the corresponding proteome intensity. Statistical analyses were performed by using a two‐tailed *t*‐test, with *p* < 0.05 and a ratio >1.2 considered statistically significant.

### In‐gel trypsin digestion

Eluted samples were analyzed by SDS‐PAGE, and bands were excised from the gel. Gel particles were washed twice with 50% ethanol for 1 h each. After washing twice with water, gels were cut to small slices, dehydrated with 100% ACN and dried in a Speed‐Vac. Reduction was performed using 10 mM DTT at 56°C for 1 h, followed by alkylation with 55 mM iodoacetamide at room temperature for 45 min in the dark. Gels were washed with 50 mM NH_4_HCO_3_, dehydrated with 25 mM in 50% ACN and dried in a Speed‐Vac. Gels were then digested with 10 ng/µL trypsin at 37°C overnight. The digested peptides were extracted using sequential elution with buffer 1 (50% ACN, 5% TFA), buffer 2 (75% ACN, 0.1% TFA) and 100% ACN. All extracted solutions were collected, dried in a Speed‐Vac and desalted by OMIX tips.

### LC‐MS/MS analysis of immunoprecipitated samples

Samples were analyzed using an Orbitrap Fusion mass spectrometer coupled with a nanospray ion source in positive mode. A 110‐min gradient was used, with 6%–30% solvent B over 90 min. The mass resolution was set to 60 K at m/z of 200 for MS1 and 7.5 K for MS2. For MS1, a m/z range of 350‐1, 600 was scanned with either a single charge or more than six charge discarded. The AGC targets were set to 3E6. For MS2, ions with intensity larger than 50,000 were isolated and sequentially fragmentized by HCD with normalized collision energy of 32%. The dynamic exclusion duration was set to 25 s.

### Western blot analysis

Cell lysates (10 µg) were analyzed using SDS‐PAGE and transferred to nitrocellulose membranes. The proteins on membrane were visualized with Ponceau staining and subsequently probed with specific antibodies. After incubation with secondary antibody, membranes were imaged using a chemiluminescence Western Blot analysis System (Clinx).

### RNA‐seq library preparation

THP‐1‐derived macrophages infected with H37Ra were washed twice with cold PBS and then lysed with Trizol. RNA purification, reverse transcription, library construction and sequencing were performed at Shanghai Majorbio Bio‐pharm Biotechnology (Shanghai, China). High‐quality RNA samples were used to construct sequencing library with the TruSeq^TM^ RNA sample preparation Kit from Illumina using 1 μg of total RNA. Briefly, messenger RNA was isolated using oligo(dT) beads and then fragmented using a fragmentation buffer. Double‐stranded cDNA was synthesized using a SuperScript double‐stranded cDNA synthesis kit. Then, the cDNA was subjected to end‐repair, phosphorylation, and “A” base addition. Libraries were size selected for cDNA target fragments of 300 bp on 2% Low Range Ultra Agarose, followed by PCR amplified. Libraries were quantified using TBS380 and sequenced with an Illumina HiSeq X Ten/NovaSeq. 6000 sequencer (2 × 150 bp read length).

### Reads mapping and alternative splicing events identification

Raw paired‐end reads were trimmed and quality‐controlled using fastp (https://github.com/OpenGene/fastp) [63] with default parameters. Then, clean reads were separately aligned to the reference genome with orientation mode using HISAT2 (http://ccb.jhu.edu/software/hisat2/index.shtml) software [64]. The mapped reads of each sample were assembled by StringTie (https://ccb.jhu.edu/software/stringtie/index.shtml?t=example) [65] in a reference‐based approach. All the alternative splice events were identified by using the recently released program rMATS [31]. Only the isoforms that were similar to the reference or comprised novel splice junctions were considered, and the splicing differences were detected as exon inclusion, exclusion, alternative 5′, 3′, and intron retention events.

To identify DEGs (differential expression genes) between two different samples, the expression level was calculated according to the transcripts per million reads method. RSEM [66] was used to quantify gene abundances, and differential expression analysis was performed using the DESeq.2 [67]. For transcriptomics, DEGs with Ratio > 1.5 and FDR < 0.05 were considered significantly different expressed genes. For splicing events, ΔPSI > 0.05 and FDR < 0.05 were considered spliced genes.

### Gene cloning, mutagenesis, and plasmids construction

The cDNA of *PLA2G7* was amplified from the total mRNA of HEK‐293T cells. The *pstP* cDNA was purchased from Beijing Tsingke Biotech Co., Ltd. The genes were cloned into pCDH plasmid with a 3X‐Flag tag added to N‐terminus. For site mutation, primers were designed to generate the site mutation sequence and integrated to pCDH. Primers used for plasmid construction and site mutagenesis are listed in Table S18. DH5α strain was used to clone plasmids with ampicillin resistance, and all constructs were verified by DNA sequencing.

### Plasmid transfection and lentiviral transduction

HEK‐293T cells were transfected using Liposomal Transfection Reagent (Yeasen) according to the manufacturer's instructions. For lentivirus packaging, cells were cultured in 10 cm dishes for 24 h to reach ~60% confluency. Then cells were incubated with Opti‐MEM (Gibco), 30 μL Liposomal Transfection Reagent (Yeasen), 6 μg recombined plasmid, 3 μg pMD2.G and 3 μg psPAX2 for 6 h. Next, cells were cultured in DMEM, and the medium was collected at 48 and 72 h and stored at 4°C. For lentivirus transduction, iBMDM and THP‐1 cells were seeded in six‐well plate of 400,000 cells per well and incubated at 37°C with 5% CO_2_ for over 12 h before transduction. Then cells were incubated with lentivirus‐containing medium supplemented with polybrene 1 μg/mL (Yeasen) for 12 h, then cultured for another 2–3 days. Positive cells were selected with 2 μg/mL puromycin for two passages.

### Coimmunoprecipitation analysis

HEK‐293T cells at ~70%–80% confluency were transfected with Flag‐PstP or Flag‐PstP‐3G plasmids using liposomal transfection reagent (Yeasen) and cultured for 24 h. Cells were lysed in IP buffer (100 mM NaCl, 20 mM Tris‐HCl, 0.5 mM EDTA, 0.5% (v/v) NP‐40, 2X protease inhibitor, pH 8.0) on ice for 30 min and sonicated (2 s on, 5 s off for 3 min). Lysates were centrifuged at 21,300 *g* for 10 min, and supernatants were precleared with 20 µL mouse IgG beads at 4°C for 4 h. Samples were then incubated overnight at 4°C for 16 h with 20 µL anti‐FLAG beads. Beads were washed seven times with 500 μL IP buffer at room temperature and collected in 20 µL of SDS loading buffer. The samples were then analyzed by LC‐MS/MS.

### PstP purification and dephosphorylation assay in vitro

PstP and PstP‐3G were purified by anti‐FLAG beads from overexpressed HEK‐293T cells. Beads capturing PstP or PstP‐3G were directly added to the reaction mixture containing analysis buffer (50 mM Tris‐HCl, 5 mM DTT, 4 mM MnCl_2_, pH 8.0) and 50 ng/µL synthesized phosphorylated peptide SRGRDS(pho)YGGPP (Shanghai Hongtide Biotechnology). The reaction was incubated with gentle shaking at 37°C for 2 h. Peptides were desalted using Ziptip, and 100 fmol peptides were analyzed by LC‐MS/MS.

### RNA isolation and reverse transcription‐polymerase chain reaction (RT‐PCR)

Total RNA was extracted using a Cell Fast RNA Extraction Kit (ABclonal) according to the manufacturer's instructions. Isolated RNA was reverse‐transcribed to cDNA in 10 µL reaction volume. Subsequently, PCR amplification was used to verify RNA spicing events using primers listed in Table S19. PCR products were analyzed on 1%–2% agarose gel and imaged using (Clinx). The results were quantified by the ImageJ software.

### Quantitative real‐time polymerase chain reaction (qPCR)

qPCR analysis was conducted using a 20 µL reaction according to the manufacturer's instructions (ABclonal). Data acquisition was performed using a Bio‐Rad system. Gene expression levels were normalized to ACTB expression. The primers used are shown in Table S20.

### Structure analysis

The predicted structures of PLA2G7 isoforms were determined using AlphaFold2 (AlphaFold2. ipynb ‐ Colab (google.com)). Multiple protein alignments and structure analyses were performed using PyMOL software.

### Bioinformatics analysis

Gene Ontology (GO) and KEGG pathway enrichment analyses were performed using WebGestalt (http://www.webgestalt.org/). When FDR value was 0, it was normalized to the minimum value. PPI analysis and sample clustering were conducted using the STRING database (version 12.0) (https://string-db.org/) with a confidence interaction score ≥0.4. Cytoscape software was used to construct the PPI clusters with score ≥4. KSEA was performed using KSEA App (https://casecpb.shinyapps.io/ksea/) [68] with PhosphoSitePlus database, setting the *p*‐value cutoff at 0.05 and substrate count cutoff at 5.

### Statistical analysis

The two‐tailed unpaired Student's *t*‐test was used to determine the statistical significance of differences between two groups. The data are presented as means ± standard error of the mean (SEM), *p < 0.05, **p < 0.01, and ***p < 0.001.

## AUTHOR CONTRIBUTIONS

**Tianxian Liu**: Writing—review and editing; writing—original draft; project administration; formal analysis. **Jun‐Yu Xu**: Conceptualization; funding acquisition; writing—review and editing. **Lei Zhao**: Writing—review and editing; project administration. **Yameng Fan**: Writing—review and editing; data curation. **Shuyu Xie**: Writing—original draft; data curation. **Ke Ma**: Writing—original draft. **Ying Zhou**: Conceptualization; investigation. **Minjia Tan**: Conceptualization; resources; funding acquisition. **Bang‐Ce Ye**: Conceptualization; funding acquisition; resources.

## CONFLICT OF INTEREST STATEMENT

The authors declare no conflicts of interest.

## 1 ETHICS STATEMENT

No animals or humans were involved in this study.

## Supporting information


**Figure S1.** H37Ra infection affects RNA splicing.
**Figure S2.** *M. smeg* infection decreased phosphorylation and disturbed RNA splicing.
**Figure S3.** H37Ra infection transcriptomic analysis and RNA splicing information.
**Figure S4.** PstP dephosphosites sequence motif and sites overlap with H37Ra infection.
**Figure S5.** Screening of upstream spliceosomes regulating PLA2G7 AS.

**Table S1.** TMT‐based proteomic data of THP‐1 after infected by *Mycobacterium tuberculosis* H37Ra.
**Table S2.** Gene Ontology biological process (GO‐BP) enrichment analysis of upregulated proteins after infected by *Mycobacterium tuberculosis* H37Ra.
**Table S3.** TMT‐based phosphoproteomic data of THP‐1 after infected by *Mycobacterium tuberculosis* H37Ra.
**Table S4.** GO‐BP enrichment analysis of proteins with significantly changed phosphosites after infected by *Mycobacterium tuberculosis* H37Ra.
**Table S5.** TMT‐based proteomic data of THP‐1 after infected by *Mycobacterium smegmatis* MC2 155.
**Table S6.** GO‐BP enrichment analysis of proteins with up regulated phosphosites after infected by *Mycobacterium smegmatis* MC2 155.
**Table S7.** TMT‐based phosphoproteomic data of THP‐1 after infected by *Mycobacterium smegmatis* MC2 155.
**Table S8.** GO‐BP enrichment analysis of proteins with significantly changed phosphosites after infected by *Mycobacterium smegmatis* MC2 155.
**Table S9.** Transcriptomic data of THP‐1 after infected by *Mycobacterium tuberculosis* H37Ra.
**Table S10.** GO‐BP enrichment analysis of upregulated genes after infected by *Mycobacterium tuberculosis* H37Ra.
**Table S11.** Alternative splicing events in THP‐1 cell after infected by *Mycobacterium tuberculosis* H37Ra.
**Table S12.** Enrichment analysis of alternative splicing genes after infected by *Mycobacterium tuberculosis* H37Ra.
**Table S13.** TMT‐based proteomic data of HEK‐293T cell after overexpressed with PstP.
**Table S14.** TMT‐based phosphoproteomic data of HEK‐293T cell after overexpressed with PstP.
**Table S15.** KEGG enrichment analysis of proteins with down regulated phosphosites after overexpressed with PstP.
**Table S16.** PstP co‐immunoprecipitation (CO‐IP) combined with LC‐MS/MS results.
**Table S17.** KEGG enrichment analysis of PstP co‐immunoprecipitated proteins.
**Table S18.** List of primers used for plasmid construction in this study.
**Table S19.** List of primers used for reverse transcription‐polymerase chain reaction (RT‐PCR) in this study.
**Table S20.** List of primers for quantitative real‐time polymerase chain reaction (qPCR) in this study.

## Data Availability

All mass spectrometry raw data have been deposited to the iProX Consortium with Project ID: IPX0007089001 (URL: https://www.iprox.cn/page/DSV021.html;?url=1707280959630Rc6J, Password: th0B). And the transcriptomics raw data have been deposited to GEO (GEO Accession viewer, Password: qzitsqwwblmppkh). The data and scripts used are saved in GitHub (https://github.com/Tianxian-Liu/Mtb-infection-affect-host-phosphorylation). Supplementary materials (figures, tables, scripts, graphical abstract, slides, videos, Chinese translated version, and update materials) can be found in the online DOI or iMeta Science http://www.imeta.science/imetaomics/.
