# Supplementary material for: Mycobacterial PstP impairs host RNA alternative splicing by dephosphorylation of spliceosome RBMX at S189
Source: IMetaOmics. 2025 Jan 9;2(1):e53. doi: 10.1002/imo2.53 (PMC12806253; doi:10.1002/imo2.53)
Supplement: Supplementary file 1 — Figure S1. H37Ra infection affects RNA splicing. Figure S2. M. smeg infection decreased phosphorylation and disturbed RNA splicing. Figure S3. H37Ra infection transcriptomic analysis and RNA splicing information. Figure S4. PstP dephosphosites sequence motif and sites overlap with H37Ra infection. Figure S5. Screening of upstream spliceosomes regulating PLA2G7 AS. [file IMO2-2-e53-s002.docx]

**Supporting Information to**

**Mycobacterial Pstp Impairs Host RNA Alternative Splicing by Dephosphorylation of Spliceosome RBMX at S189**

**Running title:** Multi-Omics analysis of Mtb-infected cells

Tianxian Liu^12#^, Jun-Yu Xu^23#*^, Lei Zhao^13^, Yameng Fan^2^, Shuyu Xie^2^, Ke Ma^2^, Ying Zhou^1^, Minjia Tan^23*^, Bang-Ce Ye^1*^

^1^ Laboratory of Biosystems and Microanalysis, State Key Laboratory of Bioreactor Engineering, East China University of Science and Technology, Shanghai, 200030, China

^2^ State Key Laboratory of Drug Research, Shanghai Institute of Materia Medica, Chinese Academy of Sciences, Shanghai, 201203, China

^3^ Zhongshan Institute for Drug Discovery, Shanghai Institute of Materia Medica, Chinese Academy of Sciences, Zhongshan, 528400, China

^#^ These authors contributed equally: Tianxian Liu, Jun-Yu Xu

*Corresponding author: [bcye@ecust.edu.cn](mailto:bcye@ecust.edu.cn) (Bang-Ce Ye); [mjtan@simm.ac.cn](mailto:mjtan@simm.ac.cn) (Minjia Tan); [jyxu@simm.ac.cn](mailto:jyxu@simm.ac.cn) (Jun-Yu Xu)

**
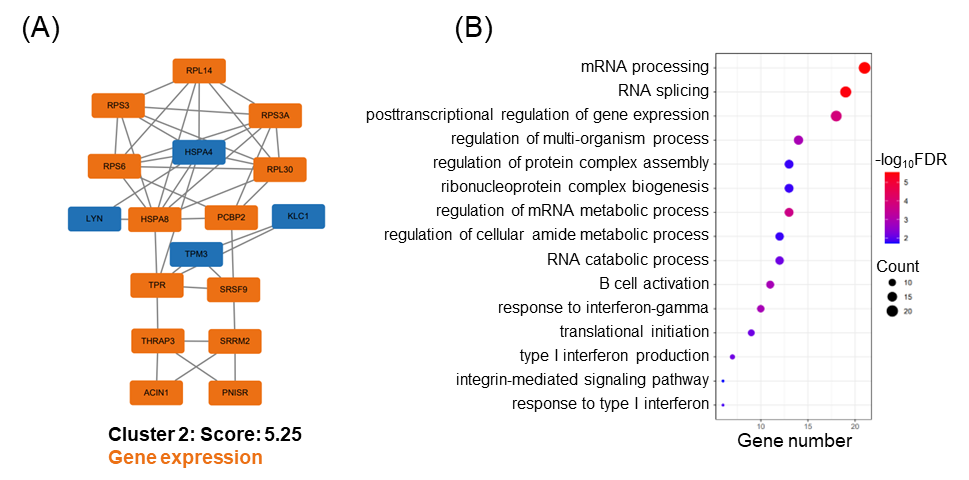
**

Figure S1 H37Ra infection affects RNA splicing. (A) Protein-protein interaction (PPI) analysis of phosphorylation down regulated proteins, cluster 2 with score 5.25. Proteins in orange are gene expression related proteins. (B) GO-BP enrichment of phosphorylation changed proteins.


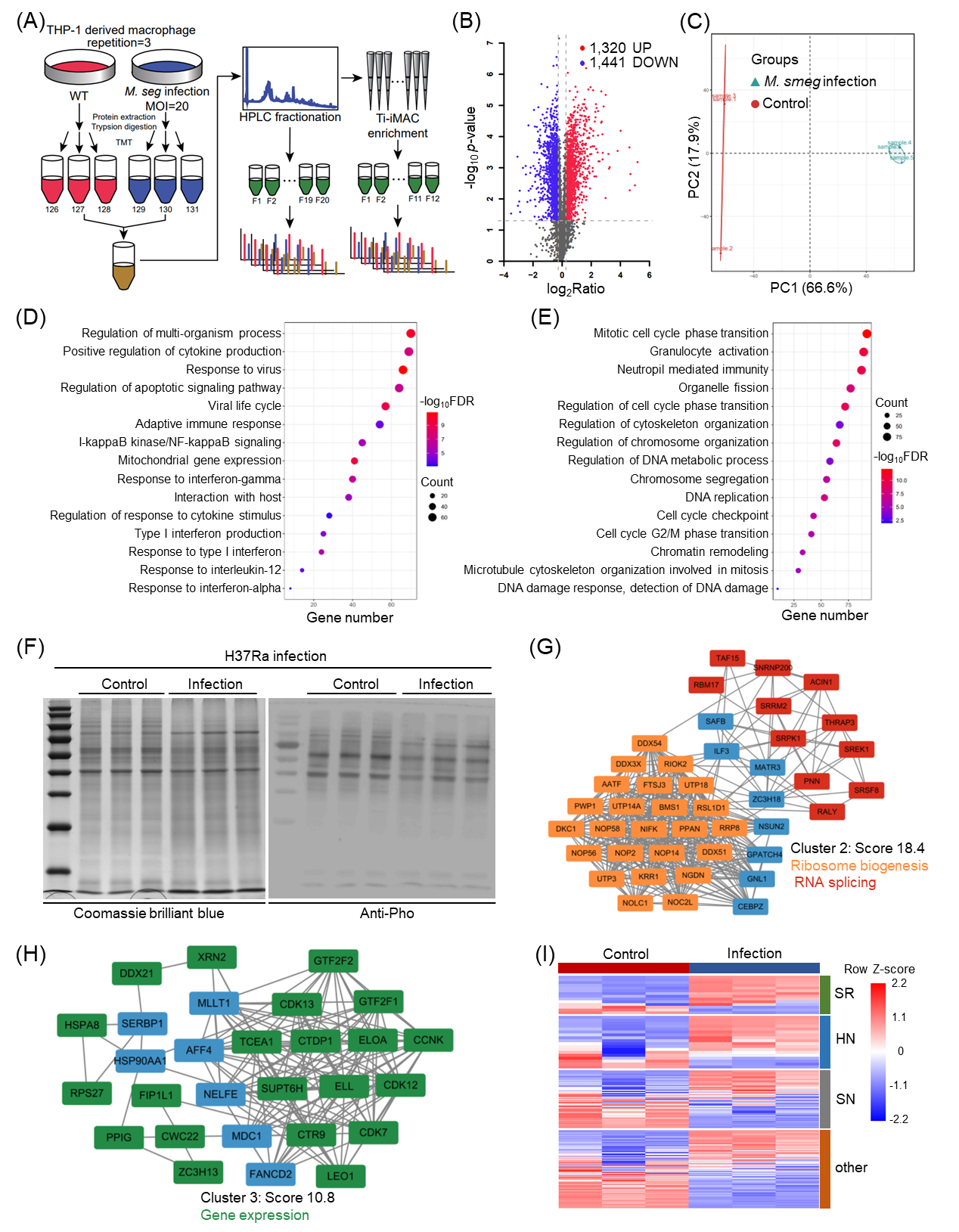


Figure S2 *M. smeg* infection decreased phosphorylation and disturbed RNA splicing. (A) Workflow of *M. smeg* infection samples TMT label and LC-MS/MS analysis. (B) Volcano plot of protein quantification after *M. smeg* infection (Infection/Control). (C) PCA of replicates in proteomics before and after infection. (D and E) GO-BP enrichment analysis of (D) up regulated proteins and (E) down regulated proteins after infection. (F) Western blot analysis of global phosphorylation changes after infected with *M. smeg*. (G and H) Protein-protein interaction (PPI) analysis of phosphorylation down regulated proteins, cluster 2 and cluster 3. (I) Heatmap of protein level changes in RNA splicing related proteins after infection.


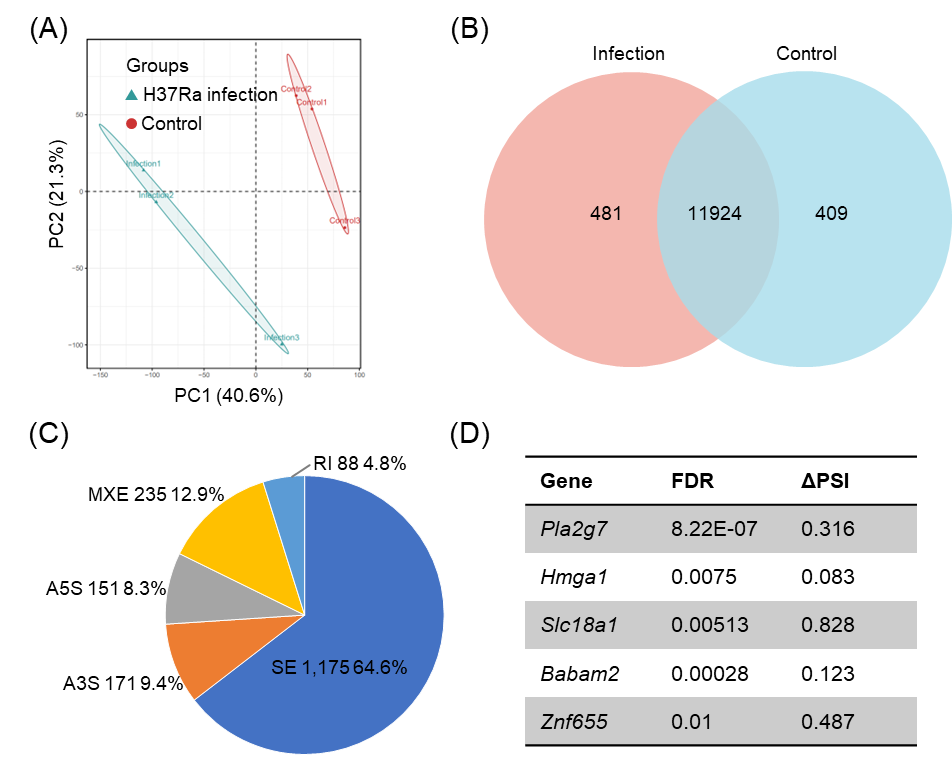


Figure S3 H37Ra infection transcriptomic analysis and RNA splicing information. (A) PCA of transcriptomic replicates before and after infection. (B) Common and specific genes translation in transcriptomics data. (C) Number and ratio of AS events in 5 splicing types. (D) ΔPSI and FDR of verified genes in transcriptomics.


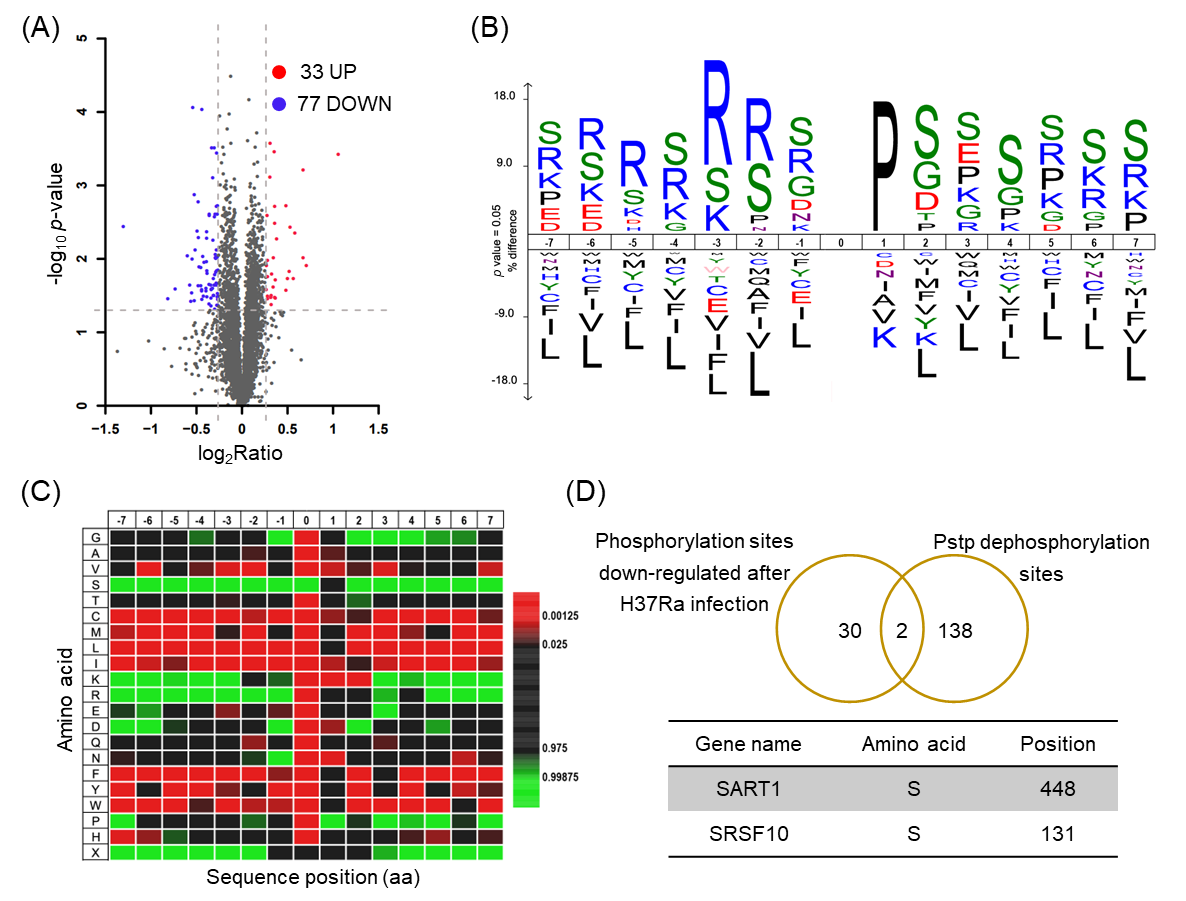


Figure S4 Pstp dephosphosites sequence motif and sites overlap with H37Ra infection. (A) Volcano plot of protein quantification after Pstp overexpression. (B and C) Motif analysis of Pstp dephosphosites arrounding sequence. (D) Overlap of H37Ra infection down regulated and Pstp overexpression down regulated splicing related proteins phosphosites.


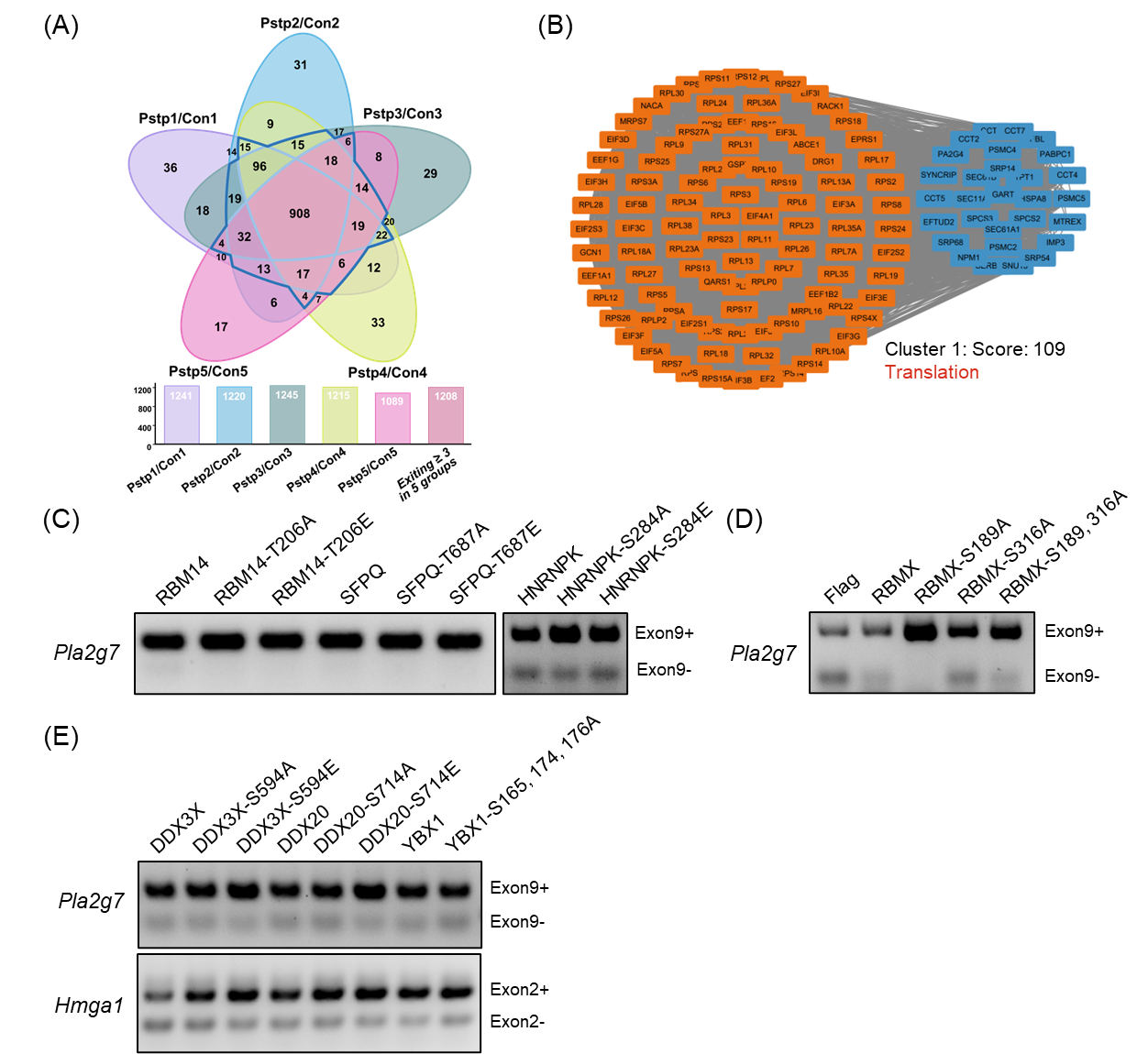


Figure S5. Screening of upstream spliceosomes regulating Pla2g7 AS. (A) Identified Pstp interaction proteins in 5 groups and overlap between each group. (B) Cluter 1 of Pstp interaction proteins PPI, score 109. (C, D and E) Upstream spliceosomes screen of Pla2g7 AS regulation proteins.
